# Emerging clinical relevance of microbiome in cancer: promising biomarkers and therapeutic targets

**DOI:** 10.1093/procel/pwad052

**Published:** 2023-11-08

**Authors:** Jia-Hao Dai, Xi-Rong Tan, Han Qiao, Na Liu

**Affiliations:** State Key Laboratory of Oncology in South China, Guangdong Key Laboratory of Nasopharyngeal Carcinoma Diagnosis and Therapy, Guangdong Provincial Clinical Research Center for Cancer, Sun Yat-sen University Cancer Center, Guangzhou 510050, China; State Key Laboratory of Oncology in South China, Guangdong Key Laboratory of Nasopharyngeal Carcinoma Diagnosis and Therapy, Guangdong Provincial Clinical Research Center for Cancer, Sun Yat-sen University Cancer Center, Guangzhou 510050, China; State Key Laboratory of Oncology in South China, Guangdong Key Laboratory of Nasopharyngeal Carcinoma Diagnosis and Therapy, Guangdong Provincial Clinical Research Center for Cancer, Sun Yat-sen University Cancer Center, Guangzhou 510050, China; State Key Laboratory of Oncology in South China, Guangdong Key Laboratory of Nasopharyngeal Carcinoma Diagnosis and Therapy, Guangdong Provincial Clinical Research Center for Cancer, Sun Yat-sen University Cancer Center, Guangzhou 510050, China

**Keywords:** microbiome, cancer, diagnosis, prognosis, therapy

## Abstract

The profound influence of microbiota in cancer initiation and progression has been under the spotlight for years, leading to numerous researches on cancer microbiome entering clinical evaluation. As promising biomarkers and therapeutic targets, the critical involvement of microbiota in cancer clinical practice has been increasingly appreciated. Here, recent progress in this field is reviewed. We describe the potential of tumor-associated microbiota as effective diagnostic and prognostic biomarkers, respectively. In addition, we highlight the relationship between microbiota and the therapeutic efficacy, toxicity, or side effects of commonly utilized treatments for cancer, including chemotherapy, radiotherapy, and immunotherapy. Given that microbial factors influence the cancer treatment outcome, we further summarize some dominating microbial interventions and discuss the hidden risks of these strategies. This review aims to provide an overview of the applications and advancements of microbes in cancer clinical relevance.

## Introduction

Microbiome can impact cancer tumorigenesis and malignant progression (Park et al., 2022), and ~13% of global cancer incidence is attributable to infectious agents (de Martel et al., 2020). Microbiome is a complex ecosystem consisting of microorganisms (bacteria, archaea, fungi, viruses, and protists), their genomes, and the surrounding environmental conditions. Despite a wide variety, current studies mainly focus on bacteria community. The majority of microbiota is located in the gastrointestinal (GI) tract, whereas only 29% of gut microbes can be captured by traditional culture-based methods according to the most recent work of the Unified Human Gastrointestinal Genome (UHGG) (Almeida et al., 2021). With the advent of next-generation sequencing (NGS) technology and culture-independent techniques, researchers can phylogenetically characterize the microbial components and quantify the diversity and abundance of microbiota (Almeida et al., 2021). Low-biomass microbial populations have been detected in other niches previously considered sterile, such as the lung, breast, liver, pancreas, prostate, and bladder (Erb-Downward et al., 2020). Further studies have identified that intratumoral bacterial and fungal composition is tumor-type specific, revealing the intrinsic association between intratumoral microbiota and cancer (Nejman et al., 2020; Narunsky-Haziza et al., 2022). The multidimensional participation in the tumorigenesis and progression of cancers qualifies the microbiome as promising biomarkers and therapeutic targets.

In this review, the diagnostic and prognostic capacities of cancer microbiome are comprehensively described. Additionally, studies as regards enhancing the therapeutic efficacy and alleviating therapeutic toxicity or side effects of commonly utilized cancer treatment are summarized. Furthermore, dominating microbial interventions and the hidden risks of these strategies are presented. We specifically highlight the clinical relevance of microbiota in cancers, aiming to direct individualized microbiota-targeted therapeutic strategies in the future.

## Clinical applications

**Figure 1.**
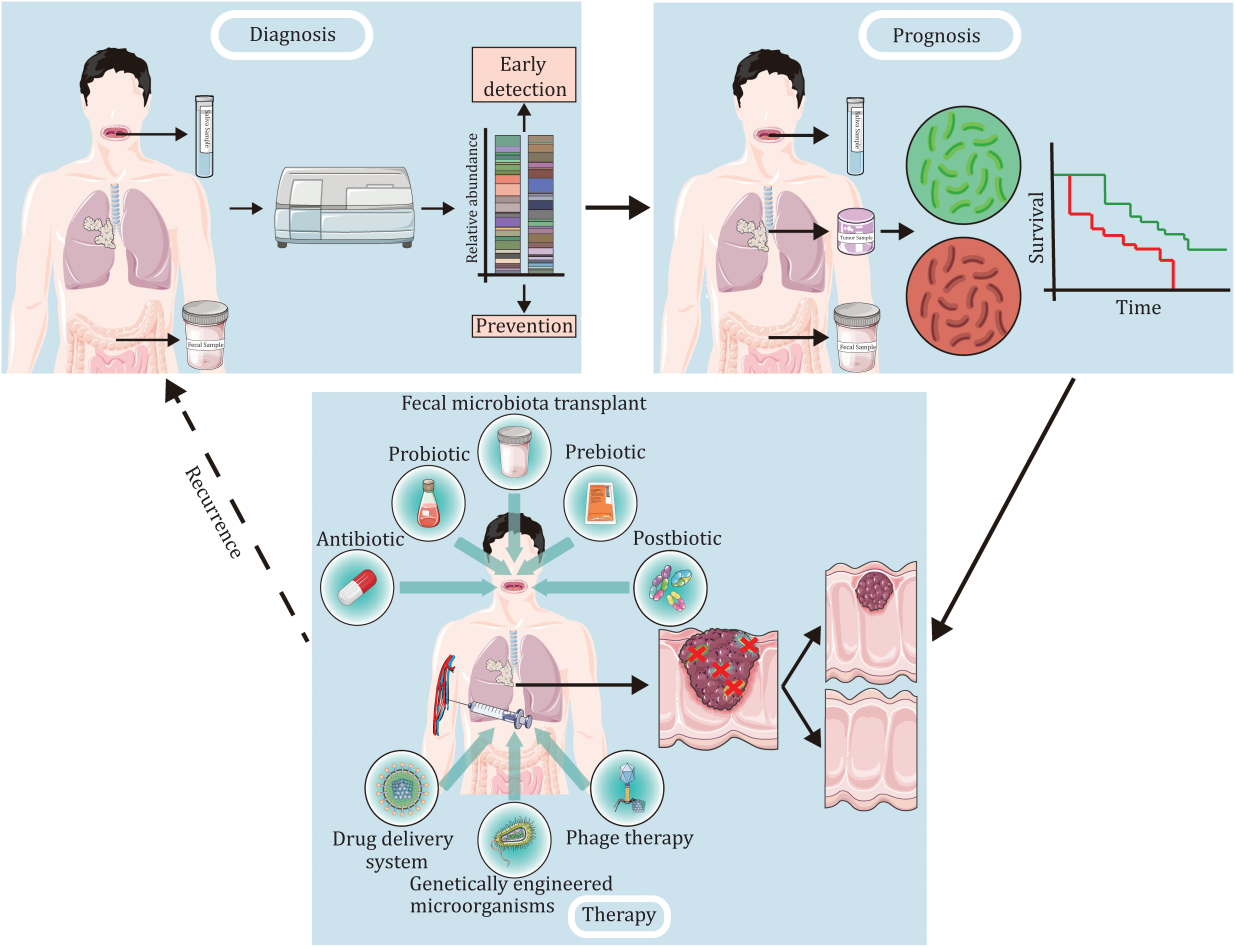
Clinical applications based on microbiome in cancer diagnosis, prognosis, and therapy.

With a deeper understanding of host–microbiota interactions, an increasing body of evidence has proven the clinical utility of tumor-associated microbiota for diagnostic, prognostic, or therapeutic purposes (Fig. 1).

### Diagnosis

In terms of diagnosis, bioinformatic analysis of microbial sequences from The Cancer Genome Atlas (TCGA) has allowed for identifying the tumor-type specific bacterial and fungal signatures across multiple cancers (Poore et al., 2020; Dohlman et al., 2022). Consistently, two large-scale studies on the intratumoral microbiota have been reported at a tissue level (Nejman et al., 2020; Narunsky-Haziza et al., 2022). Different cancer types harbor distinct bacterial or fungal composition features and ecological characteristics, indicating that intratumoral microbiome has a tremendous potential to distinguish cancer patients from healthy individuals (Nejman et al., 2020; Narunsky-Haziza et al., 2022). Therefore, the microbiota-targeted biomarkers may serve as a potential noninvasive tool for early cancer diagnosis. Two core principles for cancer screening are defined as follows: early detection refers to detecting invasive cancer early (Table 1), and prevention refers to finding and removing premalignant lesions (Table 2), both of which are critical approaches to reduce cancer burden (Bretthauer and Kalager, 2013).

**Table 1.** Microbial signatures on cancer early detection.

Cancer type	Sample	Method	Key microbial feature	Identification	Test accuracy (AUC)	References
CRC	Fecal	qPCR	*Fusobacterium nucleatum*	Healthy control versus CRC	AUC = 0.83 (0.78–0.89)	Wong et al., (2017)
			*Peptostreptococcus anaerobius*		AUC = 0.72 (0.65–0.80)	
			*Parvimonas micra*		AUC = 0.73 (0.66–0.80)	
			*F. nucleatum* and FIT		AUC = 0.95 (0.92–0.98)	
		qPCR	*F. nucleatum*		AUC = 0.850 (0.815–0.881)	Liang et al., (2021)
			*Lachnoclostridium* sp. *m3*		AUC = 0.751 (0.709–0.789)	
			*Clostridium hathewayi*		AUC = 0.678 (0.634–0.720)	
			*Bacteroides clarus*		AUC = 0.518 (0.472–0.564)	
			4 gut bacterial feature		AUC = 0.904 (0.874–0.929)	
		16S rRNA sequencing	34 gut bacterial feature, age, sex, and BMI		AUC = 0.93	Wu et al., (2021)
		Metagenomic sequencing	12 gut bacterial feature		AUC = 0.8322	Lin et al., (2022)
			5 gut fungal and 9 bacterial feature		AUC = 0.9002	
		Metagenomic sequencing	14 gut fungal feature		AUC = 0.93 (0.85–1)	Coker et al., (2019)
		Metagenomic sequencing	9 gut archaeal feature		AUC = 0.82 (0.70–0.94)	Coker et al., (2020)
		Metagenomic sequencing	22 gut viral feature		AUC = 0.802	Nakatsu et al., (2018)
		Metagenomic sequencing	27 gut bacterial feature		AUC = 0.80	Liu et al., (2022)
			20 gut fungal feature		AUC = 0.77	
			20 gut archaeal feature		AUC = 0.74	
			21 gut viral feature		AUC = 0.72	
			11 gut bacterial and 4 fungal and 1 archaeal feature		AUC = 0.83	
			175 EggNOG genes feature		AUC = 0.86	
			47 KO genes feature		AUC = 0.82	
			20 KEGG pathways feature		AUC = 0.74	
		Metagenomic sequencing	22 microbial SNV markers feature		AUC = 0.7535	Ma et al., (2021)
		Metagenomic sequencing	6 gut bacterial feature		AUC = 0.9005 (0.8703–0.9397)	Coker et al., (2022)
		GC-TOFMS	20 microbial metabolites feature		AUC = 0.8005 (0.7457–0.8554)	
			13 microbial metabolites feature		AUC = 0.7168 (0.6523–0.7813)	
			11 microbial metabolites feature		AUC = 0.6764 (0.6098–0.7431)	
		Metagenomic sequencing and GC-TOFMS	6 gut bacterial and 11 microbial metabolites feature		AUC = 0.9417 (0.9151–0.9683)	
	Oral	16S rRNA sequencing	5 oral bacterial feature		AUC = 0.7642 (0.671–0.8574)	Zhang et al., (2020a)
		16S rRNA sequencing	16 oral bacterial feature		AUC = 0.9 (0.83–0.9)	Flemer et al., (2018)
		qPCR	*F. nucleatum*		AUC = 0.841 (0.797–0.879)	Zhang et al., (2022)
	Serum	Liquid chromatography-mass spectrometry	8 microbial metabolites feature	Healthy control versus CRC and adenoma	AUC = 0.95 (0.85–1)	Chen et al., (2022b)
PDAC	Fecal	Metagenomic sequencing	30 gut bacterial feature	Healthy control versus PDAC	AUC = 0.78 (0.72–0.85)	Nagata et al., (2022)
		Metagenomic sequencing and 16S rRNA sequencing	27 gut bacterial feature		AUC = 0.84	Kartal et al., (2022)
			27 gut bacterial feature and CA19-9		AUC = 0.94	
	Oral	Metagenomic sequencing	18 oral bacterial feature		AUC = 0.82 (0.75–0.89)	Nagata et al., (2022)
HCC	Fecal	Miseq sequencing	30 gut bacterial feature	Healthy control versus HCC	AUC = 0.8064 (0.7447–0.868) Validation cohort (early HCC): AUC = 0.7680 (0.6790–0.8570) Validation cohort (advanced HCC): AUC = 0.8040 (0.7070–0.9020)	Ren et al., (2019)
ccRCC	Fecal	16S rRNA sequencing	5 gut bacterial feature	Healthy control versus ccRCC	AUC = 0.933 (0.881–0.984)	Chen et al., (2022a)
Lung adenocarcinoma	Fecal	16S rRNA sequencing	32 gut bacterial feature	Healthy control versus lung adenocarcinoma	AUC = 0.76 ± 0.08	Lim et al., (2021)
		16S rRNA sequencing	13 gut bacterial feature		AUC = 0.976 (0.954-0.998)	Zheng et al., (2020)
	Oral	16S rRNA sequencing	31 oral bacterial feature		AUC = 0.95 ± 0.03	Lim et al., (2021)
Oral squamous cell carcinoma	Oral	16S rRNA sequencing	*Streptococcus*	Healthy control versus oral squamous cell carcinoma	AUC = 0.75 (0.67–0.83)	Su et al., (2021)
			*Fusobacterium*		AUC = 0.70 (0.61–0.78)	
			*Peptostreptococcus*		AUC = 0.67 (0.58–0.75)	
			*Campylobacter*		AUC = 0.66 (0.58–0.75)	
			*Streptococcus pneumoniae*		AUC = 0.74 (0.66–0.82)	
			*F. nucleatum*		AUC = 0.66 (0.57–0.75)	
Cervical cancer	Fecal	16S rRNA sequencing	7 gut bacterial feature	Healthy control versus cervical cancer	AUC = 0.913	Kang et al., (2020)
	Vaginal	16S rRNA sequencing	*Gardnerella*		AUC = 0.953	Kang et al., (2021)
			*Streptococcus*		AUC = 0.922	
			*Finegoldia*		AUC = 0.781	
			*Anaerococcus*		AUC = 0.766	
			*Lactobacillus*		AUC = 0.719	

Abbreviations: BMI, body mass index; ccRCC, clear cell renal cell carcinoma; FIT, fecal immunochemical test; GC-TOFMS, gas chromatography coupled to time-of-flight mass spectrometer; HCC, hepatocellular carcinoma; qPCR, quantitative real-time polymerase chain reaction; rRNA, ribosomal ribonucleic acid; SNV, single nucleotide variant.

**Table 2.** Microbial signatures on cancer prevention.

Cancer types	Sample	Method	Key microbial feature	Identification	Result	References
Colorectal adenoma	Fecal	qPCR	*Fusobacterium nucleatum*	Healthy control versus colorectal adenoma	AUC = 0.59 (0.51–0.67)	Wong et al., (2017)
			*F. nucleatum* and FIT		AUC = 0.65 (0.58–0.73)	
		qPCR	*F. nucleatum*		AUC = 0.591 (0.545–0.636)	Liang et al., (2021)
			*Lachnoclostridium* sp. *m3*		AUC = 0.661 (0.616–0.704)	
			*Clostridium hathewayi*		AUC = 0.536 (0.490–0.582)	
			*Bacteroides clarus*		AUC = 0.510 (0.464–0.556)	
			4 gut bacterial feature		AUC = 0.639 (0.593–0.682)	
		16S rRNA sequencing	8 gut bacterial feature		AUC = 0.80 ± 0.07	Wu et al., (2021)
		Metagenomic sequencing	17 gut fungal feature		AUC = 0.5717	Lin et al., (2022)
			6 gut fungal and 4 bacterial feature		AUC = 0.6844	
		Metagenomic sequencing	14 gut fungal feature		AUC = 0.6-0.63	Coker et al., (2019)
		Metagenomic sequencing	14 gut bacterial feature		AUC = 0.8408 (0.7953–0.8864)	Coker et al., (2022)
		GC-TOFMS	20 microbial metabolites feature		AUC = 0.661 (0.5958–0.7262)	
			13 microbial metabolites feature		AUC = 0.6648 (0.6002–0.7295)	
			11 microbial metabolites feature		AUC = 0.6853 (0.6223–0.7482)	
		Metagenomic sequencing and GC-TOFMS	14 gut bacterial and 2 microbial metabolites feature		AUC = 0.8759 (0.8358–0.916)	
	Oral	16S rRNA sequencing	5 oral bacterial feature		AUC = 0.9594 (0.9083–1)	Zhang et al., (2020a)
	Fecal	16S rRNA sequencing	24 gut bacterial feature	Colorectal adenomas versus CRC	AUC = 0.89 ± 0.03	Wu et al., (2021)
		Metagenomic sequencing	12 gut bacterial feature		AUC = 0.8295	Lin et al., (2022)
			5 gut fungal and 9 bacterial feature		AUC = 0.8639	
		Metagenomic sequencing	6 gut bacterial feature		AUC = 0.9071 (0.8727–0.9415)	Coker et al., (2022)
		GC-TOFMS	20 microbial metabolites feature		AUC = 0.7889 (0.7339–0.8439)	
			13 microbial metabolites feature		AUC = 0.81 (0.7575–0.8625)	
			11 microbial metabolites feature		AUC = 0.7464 (0.6873–0.8055)	
		Metagenomic sequencing and GC-TOFMS	6 gut bacterial and 4 microbial metabolites feature		AUC = 0.9375 (0.9107–0.9642)	
	Serum	Liquid chromatography mass spectrometry	8 microbial metabolites feature	Healthy control versus CRC and adenoma	AUC = 0.95 (0.85–1)	Chen et al., (2022b)
IPMN	Fecal	Metagenomic sequencing	30 gut bacterial feature	IPMN versus PDAC	AUC = 0.70 (0.62–0.78)	Nagata et al., (2022)
Cervical intraepithelial neoplasia	Vaginal	16S rRNA sequencing	*Lactobacillus*	Healthy control versus cervical intraepithelial neoplasia	AUC = 0.982	Kang et al., (2021)
			*Gardnerella*		AUC = 0.857	
			Unclassified		AUC = 0.839	
			*Prevotella*		AUC = 0.812	
			*Anaerococcus*		AUC = 0.714	

Abbreviations: FIT, fecal immunochemical test; GC-TOFMS, gas chromatography coupled to time-of-flight mass spectrometer; HCC, hepatocellular carcinoma; qPCR, quantitative real-time polymerase chain reaction; rRNA, ribosomal ribonucleic acid.

#### Early detection

Early detection of cancer allows earlier treatment before an incurable state, reducing morbidity, and improving prognosis (Bretthauer and Kalager, 2013). Given that gut dysbiosis is considered a pivotal event in the occurrence of colorectal cancer (CRC), numerous studies have unearthed potential fecal biomarkers. For cost-benefit considerations, using a single microbial biomarker based for diagnosis is a viable approach, such as *Fusobacterium nucleatum* (Wong et al., 2017). But higher accuracy can be achieved with a combination of other bacterial species or existing diagnostic methods like fecal immunochemical test (FIT) (Wong et al., 2017; Liang et al., 2021). Satisfactory performances of fecal bacterial markers have also been shown for diagnosing pancreatic cancer [area under the ROC curve (AUC) = 0.78–0.94] (Nagata et al., 2022; Kartal et al., 2022), lung adenocarcinoma (AUC = 0.76–0.976) (Zheng et al., 2020; Lim et al., 2021), cervical cancer (AUC = 0.913) (Kang et al., 2020), hepatocellular carcinoma (AUC = 0.8064) (Ren et al., 2019), clear cell renal cell carcinoma (AUC = 0.933) (Chen et al., 2022a), and so on. The diagnostic performance of other diagnostic models based on non-bacterial microorganisms [such as fungal (Coker et al., 2019), archaeal (Coker et al., 2020), and viral (Nakatsu et al., 2018) features] has also been described to discriminate individuals with or without CRC. Moreover, a recent study further investigates the predictability of multi-kingdom community signatures comprising 11 bacteria (such as *F*. *nucleatum*), 4 fungi (such as *Aspergillus rambellii*), and 1 archaea (*Pyrobaculum arsenaticum*), displaying a superior diagnostic accuracy for the combination of different kingdom features with an average AUC of 0.83 (Liu et al., 2022). Except for fecal samples, researchers have developed oral microbiota signatures to distinguish patients with cancer from healthy individuals in pancreatic cancer (AUC = 0.82) (Nagata et al., 2022), lung adenocarcinoma (AUC = 0.95) (Lim et al., 2021), oral squamous cell carcinoma (AUC = 0.66–0.75) (Su et al., 2021), and CRC (AUC = 0.7642–0.9) (Flemer et al., 2018; Zhang et al., 2020a; Zhang et al., 2022). Interestingly, the presence of CRC-associated pathogens in the peripheral blood, such as *F*. *nucleatum*, *Bacteroides fragilis*, and *Streptococcus gallolyticus*, can predict a subsequent diagnosis of CRC, which embodies the possibility of intestinal dysbiosis and perturbed barrier function (Kwong et al., 2018). In addition to microbial abundance and community components, other microbial characteristics, such as microbial gene functions (Liu et al., 2022), single nucleotide variants (Ma et al., 2021), microbial metabolites in the gut (Coker et al., 2022), and serum (Chen et al., 2022b), can also be exploited as biomarkers for cancer diagnosis.

#### Prevention

Preventive cancer screening is to detect and remove precursor lesions of cancers before the malignancies (Bretthauer and Kalager, 2013). Colorectal adenomas are the main precancerous precursor lesions of CRC, and it is vital to recognize and remove colorectal adenomas at a precancerous stage to alleviate the incidence of CRC. Although *F*. *nucleatum* is pivotal for CRC and is also enriched in colorectal adenomas, it exhibits less accuracy in distinguishing adenomas from healthy controls (AUC = 0.59), which only increases to 0.65 in combination with FIT (Wong et al., 2017). A study identifies an alternative microbial biomarker, *Lachnoclostridium* sp. *m3*, showing improved diagnostic performance for adenoma than *F*. *nucleatum* (Liang et al., 2021). A newly constructed Random Forest model has achieved a significantly higher accuracy in distinguishing colorectal adenomas from non-tumor controls, with an average AUC of 0.80 in the adenoma-control model and 0.89 in the adenoma-cancer model (Wu et al., 2021). Notably, oral microbial signature shows highly diagnostic performance (AUC = 0.9594), suggesting that the evaluation of saliva microbiota emerges as a better diagnostic method for colorectal adenomas (Zhang et al., 2020a). Additionally, fecal fungal signatures can serve as a complement to fecal bacterial signatures for discriminating colorectal adenomas from healthy subjects (Coker et al., 2019; Lin et al., 2022). Integrated gut and serum microbial metabolite also shows promising diagnostic accuracy for adenomas (Coker et al., 2022; Chen et al., 2022b). Intraductal papillary mucinous neoplasm (IPMN), a premalignant condition of pancreatic ductal adenocarcinoma (PDAC), is also challenging to distinguish from PDAC via conventional biomarkers (Singhi et al., 2019). A study has examined that combining gut microbiota with serum carbohydrate antigen 19-9 (CA19-9) increases the AUC for discerning the IPMN and PDAC (Nagata et al., 2022). The vaginal-derived bacterial communities also exhibit potential capability as biomarkers to differentiate premalignant lesion cervical intraepithelial neoplasia and cervical cancer with five bacteria, including *Gardnerella*, *Streptococcus*, *Finegoldia*, *Anaerococcus*, and *Lactobacillus* (Kang et al., 2021).

### Prediction

In addition to diagnosis, researchers have found that microbial biomarkers can potentially serve as a prognostic prediction tool. Studies on microbial prognostic markers mainly focus on gut and oral microbial communities due to abundant microbial populations. Besides, the prognostic significance of microbiota in some low-biomass niches, such as the airway, urinary, and intratumoral microbiota, has also been revealed.

#### Gut and oral microbiota

Gut microbial diversity and its compositional difference have been considered to be vital factors in predicting the survival of cancer patients. For instance, a high abundance of *Alistipes*, *Faecalibacterium prausnitzii*, and *Enterobacteriaceae* is significantly associated with a favorable prognosis in PDAC. In contrast, *Ruminococcus torques* is associated with a dismal prognosis (Nagata et al., 2022). Similar to diagnostic application, fecal virome signature also has independent prognostic significance in CRC patients after adjusting for potential confounding factors (Nakatsu et al., 2018). Additionally, for cancer patients who received standardized treatment, the microbial diversity, and composition in fecal samples show a predictive ability for outcome (Sims et al., 2021; Terrisse et al., 2021). The oral cavity harbors unique microbiota and equally plays a potential role in predicting prognosis. A higher abundance of *Streptococcus* and *Megasphaera* and a lower abundance of *Haemophilus* in the oral microbiota are associated with worse outcomes for patients with lung adenocarcinoma (Lim et al., 2021). The high level of *F. nucleatum* DNA in the saliva is associated with poor survival, which serves as an independent prognostic factor for CRC patients (Zhang et al., 2022). The microbial biomarkers are also identified in the saliva of patients with PDAC (Nagata et al., 2022) and oral squamous cell carcinoma (Li et al., 2023).

#### Microbiota in low-biomass niches

Notably, since the advancement of precise detection techniques, some niches once considered sterile have been detected with low-biomass microbiota, and their prognostic value has been gradually revealed. For example, the high level of the lower airway microbiota with oral commensals, such as *Streptococcus*, *Prevotella*, and *Veillonella*, is related to worse survival (Tsay et al., 2021). As for urinary microbiota, *Herbaspirillum*, *Porphyrobacter*, and *Bacteroides* are enriched in bladder cancer patients with a high risk of progression (Wu et al., 2018). In addition to the microbiota inhabiting the external parts of tumors, increasing emphasis has been placed on intratumoral microbiota. For example, *F*. *nucleatum* is widely present in multiple tumors and is usually associated with advanced tumor stage and poor survival of various cancer patients, including CRC (Mima et al., 2016), gastric cancer (Hsieh et al., 2021), esophageal cancer (Yamamura et al., 2016), cervical cancer (Huang et al., 2020), head and neck squamous cell carcinoma (Hsueh et al., 2022), and so on. A intratumoral microbiota signature (*Pseudoxanthomonas*, *Streptomyces*, *Saccharopolyspora*, and *Bacillus clausii*) and a higher alpha diversity have been identified in PDAC patients with a long-term survival (Riquelme et al., 2019). Furthermore, the total bacterial load has potential in the prognostication, as evidenced by high intratumoral bacterial load is associated with poor prognosis in patients with nasopharyngeal carcinoma (Qiao et al., 2022). Notably, intratumoral fungal signatures that identified recently seem to be novel biomarkers that are of clinical significance, which may replace or supply those currently in use (Dohlman et al., 2022; Narunsky-Haziza et al., 2022).

### Therapy

**Figure 2.**
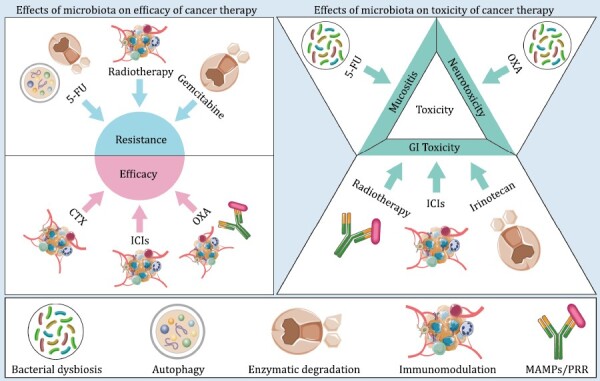
Effects of microbiome on the efficacy and toxicity of cancer therapy. Abbreviations: MAMPs, microorganism-associated molecular patterns; PRR, pattern-recognition receptor.

To date, an increasing body of evidence has shown that bidirectional influences exist between microbiome and cancer therapy, including chemotherapy, radiotherapy, and immunotherapy. For example, anticancer therapy can cause compositional and functional changes in the gut microbiome, which in turn impacts therapeutic outcomes (Wan and Zuo, 2022). Here, we summarize the current literature regarding the impact of microbiome on the efficacy and toxicity of anticancer therapy (Fig. 2). Potential applications for improving current treatment modalities by modulating microbiota, such as probiotics and fecal microbiota transplant (FMT), are also exemplified.

### Chemotherapy

#### 5-fluorocrail

Inhibiting the action of thymidylate synthase and misincorporating its metabolites into RNA and DNA have been identified as the major mechanism of 5-fluorocrail (5-FU). Microbiota has been reported to modulate 5-FU effectiveness in mice, with multiple bacterial strains capable of encoding an enzyme preTA that can interfere with 5-FU bioavailability and efficacy (Spanogiannopoulos et al., 2022). Additionally, autophagy modulated by *F*. *nucleatum* is proven to confer 5-FU chemoresistance of colon cancer cells via targeting the toll-like receptor-4 (TLR4) pathway (Yu et al., 2017). The pathological process of intestinal and oral mucositis induced by 5-FU is thought to be associated with bacterial dysbiosis (Li et al., 2017; Hong et al., 2019). The probiotics *Lactobacillus* and *Bifidobacterium* have displayed an ameliorative effect against 5-FU-induced intestinal mucositis in mouse models (Yeung et al., 2015; Kato et al., 2017). However, another study has reported that the supplementation with probiotics upon 5-FU treatment paradoxically cannot improve the therapeutic effect (Yuan et al., 2018). Therefore, it can be speculated that only specific probiotic strains are positive and synergistic for 5-FU treatment. In addition, FMT can also control adverse effects by ameliorating the gut dysbiosis induced by antibiotics or 5-FU in mouse models (Li et al., 2017).

#### Oxaliplatin

As an alkylating agent, oxaliplatin (OXA) can covalently bind DNA and form intrastrand DNA adducts, thus disrupting DNA replication and transcription. The reduced efficacy of OXA in antibiotic-treated or germ-free (GF) mice is partially due to reduced production of reactive oxygen species, in which the TLR4-myeloid differentiation factor 88 (MyD88) signaling pathway participates in this process, suggesting that microbiota may affect the tumor-inhibiting effect of OXA (Iida et al., 2013). Additionally, unlike conventional anticancer agents that are immunosuppressive, OXA can stimulate beneficial antitumor immune responses, such as causing a reduction in the proportion of Tregs and an increase in that of CD8^+^ thymocytes (Stojanovska et al., 2019). Animal experiments show that oral supplementation of *B*. *fragilis* together with OXA injection can induce higher tumor-infiltrating lymphocytes in tumors and lower CD45^+^ cells in the ileal compartment, indicating that microbiota can facilitate OXA antitumor efficacy through shaping immune profiles (Picard et al., 2021). As for toxicity, almost 90% of patients receiving OXA will develop peripheral neurotoxicity, leading to treatment withdrawal (Cheng et al., 2019). It is reported that mechanical hyperalgesia induced by OXA can be alleviated in GF mice and restoring the gut microbiome by FMT can abrogate this protection, which strongly supports the regulatory role of microbiota in OXA-induced neurotoxicity (Shen et al., 2017). Therefore, microbial intervention seems to be a potential strategy to perform synergistic antitumor effects and mitigate adverse effects caused by OXA. Interestingly, only two *Bifidobacterium bifidum* strains (*B*. *bif* K57 and *B*. *bif* K18) work synergistically with OXA to reduce tumor growth by increasing the antitumor lymphocyte population, whereas *B*. *bif* B06 and *B. bif* R71 strains show no synergistic effects (Lee et al., 2021). Another study also finds that *Bifidobacterium breve* JCM92, rather than *B*. *breve* Bb03, boosts the efficacy of OXA by enhancing antitumor immunity (Yoon et al., 2021). These studies suggest that probiotics may work synergistically with OXA by boosting host antitumor immunity in a strain-specific way. Additionally, intestinal dysbiosis and damage caused by OXA can be meliorated by modulation of gut microbiome using probiotic therapy (Yuan et al., 2022).

#### Gemcitabine

Gemcitabine, a nucleoside analog, has served as a cornerstone of systemic therapy for PDAC over a decade, which is also used in many other solid tumors, including breast, ovarian, and non-small cell lung cancer. Geller et al., (2017) have revealed that gemcitabine can be converted into the inactive form by certain intratumoral bacteria, seen mainly in *Gammaproteobacteria*, which depends on the expression of the long isoform of the bacterial enzyme cytidine deaminase (CDD). Another study has confirmed that *Klebsiella pneumoniae*, belonging to the class *gammaproteobacterial*, can facilitate chemoresistance to adjuvant gemcitabine, while quinolone treatment can reverse it and improve the survival of patients (Weniger et al., 2021). A clinical study demonstrates that lipopolysaccharide (LPS), a major component of the outer membrane of gram-negative bacteria, can predict gemcitabine efficacy in advanced PDAC as a negative biomarker (Guenther et al., 2020). In addition, CDD and pyrimidine nucleoside phosphorylase (PyNP) encoded by *Mycoplasma* can attenuate the antitumor activity of gemcitabine as well (Vande Voorde et al., 2014). Synergistic gemcitabine treatment with antibiotics may improve the treatment efficacy. For example, the selective elimination of **M*ycoplasma* and bacteria that encodes the long isoform of CDD can enhance the sensitivity of chemotherapy (Geller et al., 2017; Liu et al., 2017). Additionally, the application of probiotic *Lactobacillus* treatment also acts synergistically to enhance the anticancer effects of gemcitabine and improve the patient’s tolerance of chemotherapy (Chen et al., 2020).

#### Irinotecan

Irinotecan (SN-38G) and its active metabolite (SN-38) trigger cell death by inhibiting DNA topoisomerase I through forming a complex with DNA, ultimately disrupting DNA replication and repair. However, SN-38 also leads to damage to non-tumor cells such as blood cells and epithelial cells, which often causes a range of toxicities, including diarrhea and neutropenia. Mechanistically, the GI tract toxicity of SN-38G is mediated by β-glucuronidase (GUS) enzymes secreted by gut bacteria (Wallace et al., 2010). Once excreted into the GI tract, the inactive metabolite of SN-38G is enzymatically converted into the active form SN-38 by GUS, leading to damage of intestinal epithelial cells and diarrhea (Wallace et al., 2010). Furthermore, the activity of GUS can even serve as a predictive biomarker of irinotecan-induced diarrhea severity (Chamseddine et al., 2019). Considering the vital role of microbiota, two approaches are currently used to prevent intestinal toxicity induced by SN-38G. One strategy is to apply antibiotics, such as ciprofloxacin, to alleviate treatment-related diarrhea (Kodawara et al., 2016). Another alternative approach is a combination with GUS-specific inhibitors, which is proven to alleviate SN-38G-induced intestinal damage (Bhatt et al., 2020). Moreover, oral supplementation of probiotics, such as *Bifidobacterium animalis* subsp. *lactis SF*, is capable of enhancing the antitumor effect and weakening the intestinal and hepatic toxicity of SN-38G (Ren et al., 2022).

#### Cyclophosphamide

Cyclophosphamide (CTX) is an alkylating agent that is widely prescribed for the treatment of cancer and autoimmune diseases. CTX is metabolized by the cytochrome P450 system, which produces acrolein and the alkylating agent, phosphoramide mustard, resulting in DNA cross-linking and cell apoptosis (Hughes et al., 2018). Notably, CTX can modulate T-cell responses to exert anticancer efficacy as well, which is involved in gut microbiome. It was reported that CTX can induce intestinal dysbiosis and translocation of gram-positive bacteria in mouse models (Viaud et al., 2013). These ectopic bacteria drive the conversion of naive CD4^+^ T cells toward the Th17 pattern, while the stimulating effect of CTX is debilitated in GF mice and mice treated with antibiotics specific for gram-positive bacteria, exhibiting resistance to CTX (Viaud et al., 2013). This result emphasizes the importance of gram-positive bacteria for inducing immunogenic tumor cell death mediated by CTX. Furthermore, the gram-positive bacterial species, *Enterococcus hirae*, can translocate from the small intestine to the secondary lymphoid organs and induce positive antitumoral immune responses (Daillere et al., 2016). In addition, oral administration of *E*. *hirae* restores the efficacy of CTX in mouse models treated with antibiotics, revealing the feasibility of probiotics (Daillere et al., 2016).

### Radiotherapy

Radiotherapy is a fundamental modality in cancer treatment. Acute adverse effects occur commonly in cancer patients receiving radiotherapy, with most patients experiencing mild to moderate fatigue, skin toxicity, and mucosal injury, which causes mucositis and diarrhea (De Ruysscher et al., 2019). Currently, the tumor-associated microbiota has generated growing interest in radiosensitivity and radiation-induced complications. A systematic review has revealed that the most notable changes in the gut microbiome of patients receiving cytotoxic chemotherapy or radiotherapy are decreases in *Bifidobacterium*, *Clostridium cluster XIVa*, *F. prausnitzii*, and increases in *Enterobacteriaceae* and *Bacteroides* (Touchefeu et al., 2014). And perturbation of the intestinal microbial communities can influence the radiosensitivity in mouse models (Cui et al., 2017). Recently, Shiao et al., (2021) reveal that commensal bacteria and fungi oppositely regulate the radiobiological effects, of which the former is an essential hub of activated T cell generation following radiotherapy whereas the latter represses it by acting on macrophages to form the immunosuppressive tumor microenvironment (TME). However, little is known about the specific mechanism that how the microbiota regulates the response to radiotherapy, which is worthy of further exploration.

Acute radiation-induced GI complication is a prevalent concern that needs to be addressed. Diarrhea and mucositis occur in 80% of patients with pelvic radiotherapy and more than 90% in head and neck cancer (Peterson et al., 2015). Crawford and Gordon (2005) have investigated that GF mice are remarkably resistant to lethal radiation-induced enteritis and have less radiation-induced epithelial cell damage than normal mice with complete gut microbiome, highlighting the links between the gut microbiome and radiation injury. Antibiotic-treated mice also have significantly higher survival rates compared with controls after total body irradiation (Cui et al., 2017). The phyla *Firmicutes* and *Bacteroidetes* are the dominant bacteria in healthy gut, and the ratio of them is usually regarded as a mark of host health (Yu et al., 2017). Wang et al., (2015) have observed that the ratio of *Firmicutes* to *Bacteroidetes* is significantly altered in cancer patients with pelvic radiotherapy. A recent finding reports that the microbial diversity remains stable in patients without diarrhea and in healthy volunteers, while progressive modification in patients with diarrhea (Manichanh et al., 2008). These studies collectively suggest that typical commensal gut microbial communities play a protective role in the occurrence of adverse effects. Mechanistically, LPS has been described as a radiation protection factor for mice intestine tissue through cyclooxygenase-2 (Cox-2) with the induction of prostaglandin E2 synthesis (Riehl et al., 2000). Also, activation of TLR4 by LPS generates tumor necrosis factor-α (TNF-α), which interacts with TNF receptors on the subepithelial fibroblasts, leading to prostaglandin production and reducing radiation-induced cell death (Riehl et al., 2004). Cheng et al., (2017) reveal that polymyxin B, an antibiotic widely used to counteract the effects of endotoxin contamination, can decrease LPS-induced but increase radiation-induced mortality in mice. Studies have also demonstrated that activation of nuclear factor kappa-B (NF-κB) is involved in radiation protection from endotoxin, suggesting that TLRs may affect the response of intestinal epithelium to radio-induced injury via NF-κB pathway (Egan et al., 2004). This conclusion is further supported by the research that TLR4 agonists can attenuate radiation injury mainly through activating TLR4 and NF-κB pathway (Guo et al., 2017). These evidences reveal that strategies targeting TLRs might be protective against radiation-induced injury.

Additionally, several other strategies based on microbiota modulation have protective properties in radiation-induced injury. Preclinical studies in mice have confirmed the feasibility of *Lactobacillus rhamnosus GG* probiotic (Ciorba et al., 2012), and clinical trials show a close agreement that probiotic supplementation can reduce the incidence and severity of radiation-induced diarrhea in cancer patients (Linn et al., 2019). More profoundly, *L*. *rhamnosus* supplementation can alleviate radiation injury and enhance the crypt cell survival in a TLR-2/COX-2-dependent manner, further supporting the protective role of TLRs (Ciorba et al., 2012). Apart from probiotics, FMT has demonstrated its feasibility for alleviating radiation enteritis in mice and patients, unveiling its great potential as a safe and effective method (Cui et al., 2017; Ding et al., 2020). These studies shed new light on the radioprotective mechanisms of microbial modulation against radiation-induced mucositis, which may optimize current treatment strategies and develop novel therapeutic agents.

### Immunotherapy

Immune checkpoint inhibitors (ICIs) have exhibited potent antitumor effects against solid and hematological malignancies, which have revolutionized the oncology field. Recent studies highlight that antibiotic exposure is involved in the reduced clinical benefit of ICIs in cancer patients (Derosa et al., 2018; Routy et al., 2018). Therefore, it is essential to monitor the microbial changes in preclinical and clinical studies, thus confirming the role of microbiota in the interaction between antibiotics and ICIs. Further exploratory approaches are taken to explore the cause-effect link between microbiota and immunotherapy. Through using GF and broad-spectrum antibiotic-treated experimental mouse models, the existence of a particular group of microbiota has been proven as an essential prerequisite to exerting anticancer effects of ICIs, while the ineffective response can be overcome by reconstitution of gut microorganisms as well (Gopalakrishnan et al., 2018; Routy et al., 2018). Beyond preclinical mouse models, the impact of microbiota on the efficacy of ICIs has also gradually been well characterized in human cancer patients, in which some specific bacteria have been identified to be involved in modulating immunotherapy response (McCulloch et al., 2022). Mechanistically, it has been widely believed that the microbiota can activate innate immune mediators as well as the adaptive immune response to reprogram the immunity of TME, thus modulating immunotherapy (Lu et al., 2022). Moreover, taking probiotics can enhance the efficacy of ICIs for cancer patients (Lee et al., 2021; Yoon et al., 2021). Additionally, clinical trials have assessed the safety and feasibility of ICI responder-derived FMT and confirmed that FMT can beneficially modulate the composition of the gut microbiota and increase the clinical efficacy of immunotherapy in melanoma patients (Baruch et al., 2021; Davar et al., 2021).

Attention should be given to the autoimmune-like toxicity that arises during the ICIs therapy, termed immune-related adverse effects (IrAEs). It has been reported that the IrAEs are associated with the microbiota, particularly in colitis. The study demonstrates that the IrAEs induced by Ipilimumab occur at sites with exposure to commensal microorganisms, mostly the gut (Beck et al., 2006). Further studies have reported that gut microbial diversity (Batten et al., 2019), specific microbial abundance [such as *Bacteroidetes* (Dubin et al., 2016) and *Firmicutes* (Chaput et al., 2017)], and microbial components [such as LPS (McCulloch et al., 2022)] are significantly associated with the incidence and/or severity of IrAEs. Based on this recognition, the first case of ICIs-associated colitis successfully treated with FMT is a fairly recent attempt in this direction (Wang et al., 2018).

### Potential microbial interventions

Nowadays, a bundle of measures that capable of restoring the gut microbiome toward premorbid composition and diversity resembling healthy individuals have been applied, including probiotics, FMT, prebiotics, postbiotics, and antibiotics. The application of probiotics (Table 3) and FMT (Table 4) have been elaborated in the previous sections, which are summarized and presented in table as well.

**Table 3.** Overview of the application of probiotics in cancer therapy.

Cancer type	Intervention	Therapy regimen	Object	Purpose	Results	References
	*Lactobacillus casei variety rhamnosus* (Lcr35) *Lactobacillus acidophilus* and *Bifidobacterium bifidum* (LaBi)	5-FU	Mouse models	Reduce side-effect	Attenuated body weight loss Attenuated diarrhea and improved diarrhea scores Lower inflammatory cytokines No bacterial translocation	Yeung et al., (2015)
	*B. bifidum G9-1*	5-FU	Mouse models		Attenuated body weight loss Attenuated the severity of diarrhea Attenuated the alternation of villi and crypt cells Lower inflammatory cytokines Ameliorated dysbiosis	Kato et al., (2017)
Gastric cancer	*Clostridium butyricum*, *Bacillus mesentericus* and *Streptococcus faecalis*	OXA	Mouse models and cancer patients		Attenuated intestinal toxicity Attenuated the alternation of villus cells Ameliorated dysbiosis	Yuan et al., (2022)
	*Lactobacillus rhamnosus GG*	Radiotherapy	Mouse models		Improved crypt survival (1.95-fold, *P* < 0.01) Reduced epithelial apoptosis (33%–18%, *P* < 0.01)	Ciorba et al., (2012)
Cervical cancer	*Lactobacillus acidophilus LA-5* and *Bifidobacterium animalis* subsp. *lactis BB-12*	Radiotherapy	Cancer patients		Reduced incidence of diarrhea (53.8 and 82.1%, *P* < 0.05) Reduced mild to moderate and severe diarrhea (*P* < 0.05) Reduced usage of anti-diarrheal medication (*P* < 0.01) Reduced episodes of abdominal pain in days (*P* < 0.001)	Linn et al., (2019)
NSCLC	*B. bifidum KCTC3357* *B. bifidum KCTC3418*	OXA	Mouse models	Improve efficacy	Worked synergistically to suppress tumor growth Enhanced antitumor immunity	Lee et al., (2021)
Colon carcinoma	*Bifidobacterium breve JCM92*	OXA	Mouse models		Worked synergistically to suppress tumor growth Enhanced antitumor immunity	Yoon et al., (2021)
Fibrosarcoma, CRC, cervical cancer	*Enterococcus hirae clone 13144*	CTX	Mouse models		Worked synergistically to suppress tumor growth Restored CTX-induced anticancer immune responses	Daillere et al., (2016)
NSCLC	*B. bifidum KCTC3357* *B. bifidum KCTC3418* *B. bifidum MG731*	PD-1	Mouse models and cancer patients		Worked synergistically to suppress tumor growth Enhanced antitumor immunity Lipid-lowering effects	Lee et al., (2021)
Colon carcinoma	*Bifidobacterium breve JCM92*	PD-1	Mouse models		Worked synergistically to suppress tumor growth Enhanced antitumor immunity	Yoon et al., (2021)
PDAC	*Lactobacillus paracasei GMNL-133* and *Lactobacillus reuteri GMNL-89*	Gemcitabine	Mouse models	Improve efficacy and reduce side-effect	Lower grade of pancreatic intraepithelial neoplasia formation Lower levels of liver enzymes (AST/ALT)	Chen et al., (2020)
Colon adenocarcinoma	*B. animalis* subsp. *lactis SF*	Irinotecan	Mouse models		Worked synergistically to suppress tumor growth Enhanced antitumor immunity Attenuated diarrhea and immunosuppression Ameliorated dysbiosis and increased the abundance of anti-inflammatory flora	Ren et al., (2022)

Abbreviations: ALT, alanine aminotransferase; AST, aspartate aminotransferase; NSCLC, non-small cell lung cancer.

**Table 4.** Overview of the application of FMT in cancer therapy.

Cancer type	Intervention	Therapy regimen	Object	Purpuse	Results	References
	FMT	5-FU	Cancer patients	Reduce side-effect	Attenuated body weight loss Attenuated the shortening of colon Ameliorated dysbiosis	Li et al., (2017)
	FMT	Radiotherapy	Mouse models		Increased the survival rate of irradiated animals Elevated peripheral white blood cell counts Improves GI tract barrier function and epithelial integrity Ameliorated dysbiosis Retained the gene expression profile of the small intestine Enhanced angiogenesis without accelerating tumor growth	Cui et al., (2017)
	FMT	Radiotherapy	Cancer patients		Ameliorated rectal hemorrhage, fecal incontinence, diarrhea and abdominal and rectal pain Reduction in Radiation Therapy Oncology Group (RTOG/EORTC) late toxicity grade from baseline Ameliorated dysbiosis	Ding et al., (2020)
Urothelial carcinoma, prostate cancer	FMT	CTLA-4 and PD-1 CTLA-4	Cancer patients		Complete resolution of clinical symptoms Attenuated mucosal inflammation and ulceration Ameliorated dysbiosis	Wang et al., (2018)
Metastatic melanoma	FMT	PD-1	Cancer patients	Improve efficacy	Overcame resistance to anti-PD-1 Longer median progression-free survival Ameliorated dysbiosis Enhanced antitumor immunity	Baruch et al., (2021)
Melanoma	FMT	PD-1	Cancer patients		Overcame resistance to anti-PD-1 Ameliorate dysbiosis Enhanced antitumor immunity Lower inflammatory cytokines	Davar et al., (2021)

Abbreviations: CTLA-4, cytotoxic T lymphocyte associated protein 4.

#### Prebiotics

Prebiotics are defined as substrates that are selectively utilized and fermented by host microorganisms to confer a health benefit (Gibson et al., 2017). Prebiotics exert their function by stimulating the growth of beneficial host microorganisms, such as *Bifidobacterium* or certain species thought of as butyrate producers (Gibson et al., 2017). As these genera are usually considered probiotics, this approach provides a commonality between probiotics and prebiotics. It thus gives rise to the possibility that some functional prebiotics may facilitate cancer therapy by modulating the underlying ecological processes of microbial structure and function. For example, supplementation with *Lycium barbarum* polysaccharides can promote the production of short-chain fatty acids (SCFAs) and increase the relative abundances of *Bacteroidaceae*, *Lactobacillaceae*, *Prevotellaceae*, and *Verrucomicrobiaceae*, which were positively associated with immune traits, thus improving the efficacy of chemotherapy (Ding et al., 2019). So far, some preclinical studies on applying prebiotic supplements to remodel the gut flora structure for preventing and treating cancer have been carried out, such as isomaltooligosaccharides (Chen et al., 2022c). Despite this remarkable potential, there is still no firm data supporting the use of prebiotics to fight against cancer in the clinical patient population.

#### Postbiotics

Postbiotics refer to the metabolites capable of conferring health benefits from the metabolic activity of microbiota, which is a newly emergent research field and remains to be explored (Salminen et al., 2021). Microbial fermentation is considered a natural way to provide a variety of postbiotics. The metabolic product SCFAs represents a prototypical example, which has been demonstrated to have anticancer activity in cell cultures and animal models of cancer (Chen et al., 2019). Additionally, it has been reported that SCFAs are linked to positive anti-programmed cell death 1 (PD-1)/programmed cell death ligand 1 (PD-L1) response across different GI cancer types (Peng et al., 2020). Tryptophan metabolites derived from gut microbiota, such as indoles (Hezaveh et al., 2022), indole-3-acetic acid (Tintelnot et al., 2023), and indole-3-lactic acid (Han et al., 2023; Zhang et al., 2023), have been reported to have great potential as postbiotic supplements. Intriguingly, a recent study demonstrates that intratumoral *Lactobacillus reuteri* can release dietary tryptophan catabolite indole-3-aldehyde to promote interferon-γ (IFN-γ) producing CD8^+^ T cells and facilitate the ICIs treatment efficacy (Bender et al., 2023).

#### Antibiotics

In fact, the elimination of carcinogenic microbes by antibiotics has immense value in the prevention and treatment of cancers. *Helicobacter pylori* infection is a major risk factor for the carcinogenesis of gastric cancer, and its eradication can significantly reduce the incidence of gastric cancer (Yan et al., 2022). Some studies have reported that the addition of antibiotics treatment can ameliorate the therapeutic resistance induced by microbiota and improve the efficacy of cancer therapy (Geller et al., 2017). Wang et al., (2023) develop liposomes loaded with an antibiotic silver-tinidazole complex to eliminate the tumor-associated bacteria in primary tumor site and metastatic lesions. Interestingly, the elimination of bacteria can generate microbial neoantigens that shared homologous epitopes with the host, thus eliciting antitumoral immunity (Wang et al., 2023).

#### Phage therapy

Considering the potential carcinogenic risks of dysbiosis induced by the broad-spectrum and low-specificity germicidal action of conventional antibiotics, novel approaches targeting deleterious or pathogenic microorganisms may play a better role in cancer therapy. Indeed, the phages are considered noninfective to humans due to their high specificity for host bacteria, making them represent a viable antibiotic alternative (Principi et al., 2019). Dong et al., (2020) have screened a specifically *F*. *nucleatum* binding M13 phage that can achieve specific clearance of *F*. *nucleatum* and remodel the TME to augment systemic antitumor immunity. Additionally, modified phages can be designed to carry chemotherapeutic drugs and provide controlled release of the drug at the tumor site by targeting cancer-residing bacteria. For example, the phage that specifically lysed *F*. *nucleatum* has been designed to combine with irinotecan-encapsulated dextran nanoparticles, which are released and accumulated in the TME, thus improving the chemotherapy responses and reducing the systemic side effects of irinotecan treatment (Zheng et al., 2019). Notably, using only a single phage may develop resistance to some bacterial pathogens, which may be effectively improved by using different phage mixtures. Federici et al., (2022) have designed a lytic five-phage combination targeting *K*. *pneumoniae* in avoiding resistance. Herein, these studies propose that engineering phages hold tremendous promise for clinical applications.

#### Drug delivery system

Specific microbes can be designed to target the hypoxic tumor tissues precisely for their unique properties, such as hypoxia tropism (Wang et al., 2019). Photothermal therapy (PTT) has emerged as a new flourishing in clinical cancer treatment, which relies on photothermal agents to convert the energy of near-infrared light to heat, inducing thermal ablation of cancer cells. However, its applications are somewhat restricted by the non-specific uptake of photothermal agents in nontumoral tissues and cancer cells. Now, bacteria-assisted strategies based on *Escherichia coli* (Wang et al., 2019) or *Salmonella* (Chen et al., 2018) have been proposed recently for precise PTT and witnessed some exciting results. Such microbial hypoxia-targeted characteristics also can be used in photodynamic therapy approaches, another kind of novel noninvasive cancer treatment (Zhu et al., 2021). Another method targeting the tumor sites is exhibited by *Listeria* spp., which can infect myeloid-derived suppressor cells (MDSCs) and deliver the bacteria selectively to the tumor sites, where they spread from MDSCs into tumor cells (Chandra et al., 2013). They are spared from clearance and selectively survive in tumors with the help of the MDSCs and immunosuppressive TME (Quispe-Tintaya et al., 2013). Therefore, *Listeria* spp. can also be designed as a tumor-targeting delivery vector to deliver anticancer substances (Quispe-Tintaya et al., 2013). In conclusion, these studies provide new insight for developing engineered microbial vectors to deliver adjuvant formulations and drugs, broadening the treatment prospects for cancer patients.

#### Genetically engineered microorganisms

Gene therapy holds great promise for the treatment of cancer diseases, and different viral and non-viral gene delivery systems have been used for gene therapy. For example, the intestinal probiotic *E*. *coli* Nissle 1917 has been engineered as a targeted transport vector carrying anticancer genes to tumor hypoxic regions, such as tumor suppressor azurin (Zhang et al., 2012), p53, and/or anti-angiogenic factor Tum-5 (He et al., 2019), followed by high copy amplification and efficient expression of these anticancer genes, inducing cancer-killing effect and suppressing tumor growth. Recently, Chen et al., (2023) seek to engineer a *Staphylococcus epidermidis* strain to express melanoma tumor antigens, which can elicit a wide range of antigen-specific immune cell responses and synergize with ICIs treatment. In addition, genetic modification of metabolically related genes can alter biosynthesis and metabolism in microorganisms, thus exerting synergic or additive therapeutic effects in analogy with “Prebiotics” or “Postbiotics”. The genetic engineering of the arginine inhibitory gene in *E*. *coli* Nissle 1917 can alter the concentration of l-arginine in tumors, thus enhancing the efficacy of PD-L1 immunotherapy (Canale et al., 2021).

## Discussion

It is undeniable that many preclinical and clinical studies have provided mechanistic and supporting evidence that microbiota is an essential regulator to cancer, with an increasing appreciation of the role of microbiota in cancer occurrence and development. This review has emphasized the emerging diagnostic and prognostic role of microbiome-derived personalized data. In addition, another exciting aspect of host–microbiota interaction, i.e., the impact of microbiota on cancer treatment, is summarized, which may provide potential therapeutic strategies and partially revolutionize oncotherapy. Since microbiota is tremendously diverse and microbial research technologies are limited, there is still a lack of literature in this field. Some specific problems still need to be further explored and discussed.

### Advantages and challenges of microbial biomarkers

Existing clinical biomarkers present certain limitations in accessibility, specificity, and sensitivity, which has given rise to the need for developing novel biomarkers to replace or supply those currently in use. For the clinical application of microbial biomarkers, the primary and preferred method for cancer diagnosis and prognosis prediction is the collection of fecal and salivary samples, which is a noninvasive, economical, and user-friendly method that is easy to perform compared with other clinical examination items like colonoscopy. Beyond that, integrating complementary biomarkers from the host (patient characteristics, FIT, tumor markers) and the microbiota has already demonstrated higher accuracy and efficacy. Based on these considerations, building an accurate microbiome-based assessment regimen may help stratify cancer patients with different severity and improve risk-adapted treatment strategies, thus decreasing cancer mortality (Wong et al., 2017; Wu et al., 2021; Kartal et al., 2022).

To identify microbial biomarkers, interindividual differences are important issues that should be comprehensively considered, including genetic background, diet, lifestyle habits, health condition, physical activity, regional variations, etc., all of which can affect the microbiota composition and diversity (He et al., 2018; Manor et al., 2020). Among them, a large-scale study characterizing 7,009 individuals from 14 districts within one province in China shows that regional variations display the strongest associations with microbiota variations. These regional variations limit the extrapolations of some diagnostic models between different districts, suggesting that it is essential for clinical investigators to clearly illustrate the information of training disease models that generate reference data (He et al., 2018). To overcome this challenge, more common microbial biomarkers should be developed in different ethnic groups of cancer patients to derive the optimal diagnostic and prognostic model across populations.

### Limitations of microbial research technology

Currently, the microbiome databases are primarily obtained by 16S rRNA sequencing and shotgun metagenomic sequencing. Several challenges hinder the translation of microbial biomarkers into clinical practice, including the standardization of sampling techniques and data analysis, and validation cohorts. Importantly, it should be noted that the microbial biomass of many tumor-associated ecological sites is relatively low, and contaminating DNA can be problematic in both PCR-based 16S rRNA gene surveys and shotgun metagenomics (Salter et al., 2014; Eisenhofer et al., 2019). Therefore, when profiling the intratumoral microbiome, it is critical to take multiple measures to avoid, or at least reduce, any possible contamination, such as adding negative and positive sequencing controls, randomizing samples and treatments, critically assessing and reporting contributions of contamination during analysis, etc. (Salter et al., 2014; Eisenhofer et al., 2019). Currently, several technologies targeted these issues have been developed. 5R 16S rDNA sequencing method has been applied in formalin-fixed paraffin embedded samples, which increases the coverage and resolution of the detection of bacterial species (Nejman et al., 2020). Notably, Nino et al., (2022) have successfully developed a new single-cell RNA-sequencing method combining 16S rRNA sequencing, termed invasion–adhesion-directed expression sequencing (INVADEseq), to reveal spatial, cellular, and molecular interactions of intratumoral microbiota and the host. Therefore, the integration of multi-omics technologies and their application to the microbiome field will further shed new light on the interaction with the microbiome in the spatial and temporal scales of tumor development.

### Probiotics safety

It should be noted that there are some safety concerns about microbiota modulation strategies with live microorganisms, such as probiotics. Cancer patients are frequently at risk of immunosuppression due to cancer and treatments, which are more likely to experience higher infection rates, such as bacteremia and sepsis (Pique et al., 2019). In addition, other potential side effects also require awareness and attention, including initiation of an excessive inflammatory response, colonization of foreign pathogenic strains, translocation of live bacteria into local tissues, and the transmission of resistance genes between bacterial populations (Pique et al., 2019). Some case reports have described the adverse events associated with using live probiotics, such as septicemia, pneumonia, meningitis, endocarditis, and abscess (Deshpande et al., 2018). Therefore, the risks and the risk/benefit ratios for each probiotic strain demand careful evaluation in clinical practice. Given that only some specific probiotic stains are reported to elicit beneficial effects, it is necessary to screen and identify the strains that truly play a role in therapy (Lee et al., 2021; Yoon et al., 2021).

### Prebiotics/Postbiotics concern

Prebiotics and postbiotics do not contain live microorganisms, which seems to offer safer efficacy with a lower risk of adverse effects. A study observes that sufficient dietary fiber intake seems to benefit more than probiotic use in cancer patients with ICIs treatment (Spencer et al., 2021). But gut microbial responses to dietary prebiotics vary individually, probably due to different fermentative pathways driven by distinct microbial compositions (Holmes et al., 2022). As for postbiotics, some studies have proposed that some postbiotics have both pro- or anti-tumorigenic effects, depending on the circumstance in which they operate. SCFAs may increase the risk of hepatocellular carcinoma under specific dietary conditions (Singh et al., 2018), despite the anticancer activity mentioned above (Chen et al., 2019). Such a paradoxical phenomenon reminds us that the operational circumstance that postbiotics can perform their anticancer activities still needs to be elucidated. In addition, considering the huge number and complex types of metabolites produced by microbiota, it represents a tremendous practical challenge to isolate, enumerate, and identify specific postbiotics.

### FMT-related adverse events

An extensive systematic review of adverse events for FMT (*n* = 4,241 patients) has reported that the overall incidence of FMT-related adverse events was 19%, the majority of which were GI complications, such as diarrhea (10%) and abdominal discomfort/pain/cramping (7%) (Marcella et al., 2021). Only 1.4% of patients undergo FMT-related serious adverse events, including infections and deaths (Marcella et al., 2021). However, these original data are derived from a population with various disease groups but none specifically for cancer patients, and thus the clinical utility and consequence of FMT in cancer therapy remain poorly defined. In a more rigorous clinical study, it is essential to investigate the clinical efficacy and adverse effects of FMT for cancer patients. Interestingly, only the patients with mucosal barrier injury develop FMT-related serious adverse events (Marcella et al., 2021). This suggests that each patient may need to undergo colonoscopy examinations before and after FMT treatment to reduce the likelihood of adverse events and minimize their effects. Nowadays, to rectify the deficiencies, the modified FMT has sprung up, termed washed microbiota transplantation (WMT). The metagenomic NGS and metabolomics analysis have confirmed that increasing types and amounts of viruses and metabolites with pro-inflammatory effects can be removed during the washing process, which further improves the safety of WMT (Zhang et al., 2020b). For the first time, evidence has been provided to support the fact that WMT was safer, more precise, and more quality-controllable than the crude FMT by manual (Zhang et al. 2020b). Additionally, a study recently proposes that recipient factors, not donors, drove post-FMT species-specific strain dynamics (Schmidt et al., 2022). This reveals that rigorous policies are also needed to screen the FMT recipients to ensure patient safety and donors.

### Antibiotic-related concern

Until now, the application of antibiotics in cancer therapy is still controversial. On the one hand, specific antibiotic treatments can suppress cancer development arising from the microbial infections or dysbiosis (Ma et al., 2018), and reverse therapy resistance induced by microbiota (Geller et al., 2017; Liu et al., 2017; Weniger et al., 2021). On the other hand, due to the indiscriminate impacts on the indigenous microbiota, antibiotic treatments can potentially disturb the gut ecosystem, resulting in a loss of diversity and substantial changes in microbial community composition (Watanabe et al., 2021), and may also reduce the treatment efficiency of chemotherapy (Viaud et al., 2013; Daillere et al., 2016), radiotherapy (Cui et al., 2017), and immunotherapy (Derosa et al., 2018). Frequent overuse and misuse of antibiotics, especially broad-spectrum antibiotics, can induce antibiotic resistance of bacteria, leading to diminished antibiotic efficacy, and a higher risk of infection (Pulingam et al., 2022). Therefore, with knowledge of such contradictory properties, recommendations are proposed to restrict the duration and application of broad-spectrum antibiotics and invent antibiotics with a narrow-spectrum activity, even selectively targeting specific pathogens or pathobionts. Whether cancer patients would benefit from antibiotic treatment needs further validation by more clinical trials as well.

### Engineered microorganisms-related concern

Likewise, as novel approaches for cancer treatment, it is important to assess the safety of engineered live bacterial and phage therapeutics. The predominant concern is the triggering inflammatory responses induced by activated innate and adaptive immunity while these engineered microorganisms are released into the body. For example, colitis can be exacerbated through activating phage-specific and non-specific IFN-γ mediated immune responses despite the fact that bacteriophages can target specific invasive *E*. *coli* and suppress intestinal tumor growth (Gogokhia et al., 2019). Therefore, whether the immunogenicity of these engineered microorganisms can activate the immune responses and affect the efficacy or promote the development of cancer needs further investigation, and efforts are required for human trials. Additionally, it should be noted that the recombinant DNA from the engineered microbiota may be horizontally transmissible to other native microbes after being released into human intestines or the natural environment (Yadav and Chauhan, 2022). Special containment is required to confine the engineered microorganisms, such as kill switches (Stirling et al., 2017) and synthetic auxotrophy (Mandell et al., 2015; Rovner et al., 2015). These biocontainment techniques aim to prevent the unintended growth of engineered microorganisms when they escape from the specific clinical scenarios.

## Conclusion

In conclusion, tumor-associated microbiota plays a complex role in the initiation and development of tumors. Although the oncogenic mechanisms of microbiota have been popularly studied in the available literature, more extensive and in-depth researches are still needed to further elucidate. Importantly, the hot research field of cancer has focused on the gut microbiota, resulting in a lack of understanding of microbiota in other niches and host–microbiota interactions in different cancer types, which can be the potential future direction. In addition, despite numerous controversies in this field, it must be admitted that tumor-associated microbiota has great potential to enter the clinical translation. As the basis for developing and improving clinical practices, this may create novel strategies to offer diagnostic, prognostication, and therapeutic value for cancer patients.

## Data Availability

Not applicable.
